# Corrosion Characteristics of Silicon Carbide and Silicon Nitride

**DOI:** 10.6028/jres.098.040

**Published:** 1993

**Authors:** R. G. Munro, S. J. Dapkunas

**Affiliations:** National Institute of Standards and Technology, Gaithersburg, MD 20899-0001

**Keywords:** ash, ceramics, coal, corrosion, silicon carbide, silicon nitride, slag

## Abstract

The present work is a review of the substantial effort that has been made to measure and understand the effects of corrosion with respect to the properties, performance, and durability of various forms of silicon carbide and silicon nitride. The review encompasses corrosion in diverse environments, usually at temperatures of 1000 °C or higher. The environments include dry and moist oxygen, mixtures of hot gaseous vapors, molten salts, molten metals, and complex environments pertaining to coal ashes and slags.

## 1. Introduction

The present work is a review of the substantial effort that has been made to measure and understand the effects of corrosion with respect to the properties, performance, and durability of various forms of silicon carbide and silicon nitride. These materials are the primary candidates for the materials to be used in the development of heat exchangers for the Department of Energy’s Combustion 2000 program. The review encompasses corrosion in diverse environments, usually at temperatures of 1000 °C or higher. The environments include dry and moist oxygen, mixtures of hot gaseous vapors, molten salts, molten metals, and complex environments pertaining to coal ashes and slags.

The potential applications of advanced structural ceramics have been widely appreciated for several decades [1]. Characteristics such as high maximum use temperature, strength retention at high temperature, and chemical stability have held forth tantalizing possibilities [2,3] for more efficient engines [4], heat exchangers and recuperators [5,6] and for more durable electronic packaging [7] and chemical processing components [8]. The primary barriers that have impeded [9] the widespread development of these applications have been the susceptibility of structural ceramics to brittle fracture [10] and environmentally sensitive corrosion behavior [11], including the subsequent effects of corrosion on the thermal and mechanical performance of ceramics. The former barrier has received, by far, the greater amount of attention [12]. The corrosion of advanced ceramics, however, has proven to be a complex problem and has been recognized as a critical consideration in attaining high efficiency applications [13].

**Fig. 1:**
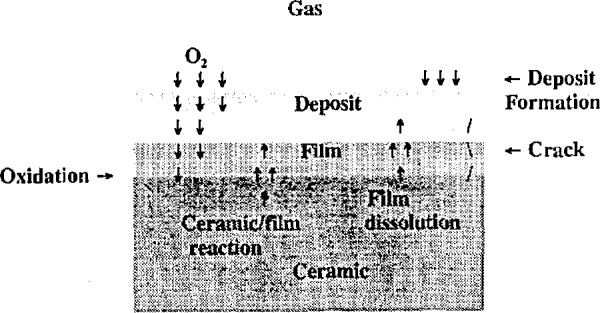
Schematic illustration of various mechanisms involved in the corrosion of a ceramic material.

A schematic illustration of the corrosion process is shown in Fig. 1. The bulk ceramic material usually may be assumed to have a film or scale on its surface that differs chemically from the bulk material. For either silicon carbide or silicon nitride, the film is usually a critically important silica layer. The bulk ceramic and its surface scale form a solid body that is exposed to a hot, flowing, gaseous environment which may also contain fine particulate matter and from which a deposit may form on the solid body. This deposit may exist in a crystalline, glassy, or molten form at the principal exposure temperature.

In this simple schematic, the corrosion process already can be seen to involve the possibility of a complex series of chemical reactions whose time-dependent course may be influenced by thermal and mechanical properties of the substrate, as well as the chemical composition of the environment. The diffusion of oxygen from the gaseous region to the ceramic, where oxidation can replenish the protective scale, may be dependent on the nature of the deposit, its density, and its thickness. Likewise, the direct and deleterious reaction between the deposit and the bulk ceramic may become possible through the formation of cracks in the protective film as a result of thermal stress gradients, the dissolution of the film in a molten deposit, and localized stress enhancement resulting from the evolution of gaseous reaction products at the interface of the ceramic and film.

The process illustrated by Fig. 1 represents the current view of the corrosion of silicon carbide quite well. For silicon nitride, the oxide scale is actually a double or duplex layer in which an intermediate phase of silicon oxynitride occurs between the outer silica scale and the silicon nitride substrate [14]. Details of the contributing and competing reactions, the formation kinetics, and the physical consequences of the corrosion process are reviewed in the following sections.

## 2. SiC

The reaction sequences for the corrosion of a material may be strongly affected by either the major or the minor components of its composition, and the rate of corrosion may be affected by the microstructure of the material. The composition determines what chemical species are available for reaction, and the structure determines how accessible the species are for the potential reactions. Consequently, understanding the corrosion of a material presupposes an understanding of the composition and structure of the material.

Crystalline silicon carbide exists in a large variety of polymorphic forms, or polytypes, that are broadly divided into two classes, α-SiC and β-SiC. The β-SiC class has a cubic structure, while the α-SiC class consists of hexagonal and rhombohedral noncubic structures. All of the varieties of SiC are formed by hexagonal layers of silicon atoms and hexagonal layers of carbon atoms arranged in various stacking patterns that result in each silicon atom being surrounded tetrahedrally by four carbon atoms, and each carbon atom being surrounded tetrahedrally by four silicon atoms. Specific varieties are commonly designated by a notation that (1) specifies the number of layers in the repeating unit and (2) indicates the symmetry of the crystallographic cell by a letter: C for cubic, H for hexagonal, and R for rhombohedral. The forms frequently noted in the literature include the cubic form, 3C of the β-SiC class, and the noncubic forms 2H, 6H, and 15R of the α-SiC class.

Powders in the β-SiC form may be produced at relatively low temperature, 1500–1600 °C, using polycarbosilanes and polyborosiloxanes in polymer conversion reactions [18], gaseous mixtures of silane, hydrogen chloride, propane, and hydrogen in vapor phase reactions [19], or low-temperature carbothermic reduction of silica and carbon with boron and titanium as additives [20]. The production of α-SiC is usually conducted at temperatures above 2500 °C and is most commonly produced using the carbothermic method known as the Acheson process [21]. Gas phase synthesis can also be used to produce α-SiC. At temperatures above 2000 °C, β-SiC may convert to α-SiC.

Bulk SiC ceramics are usually derived from SiC powders using various processing stages. As a result, SiC ceramics are generally polycrystalline materials that may consist of more than one polytype of SiC plus additional material phases resulting from the use of sintering aids or other components of the starting powders. The processing of the powders usually involves sintering, hot pressing, or reaction bonding. The sintering or hot pressing of SiC commonly requires the use of sintering aids such as boron or aluminum or their oxide, nitride, or carbide compounds. In the reaction bonding technique, carbon, silicon metal, silicon nitride, or other powders may be mixed with silicon carbide powder and allowed to react at high temperature. The resulting ceramic is generally a multiphase material that also may have a bimodal SiC grain size distribution.

It is evident that silicon carbide materials that are nominally the same may have significantly different impurities, grain size distributions, grain boundary phases, and types and extent of porosity. These differences are significant in the context of corrosion because of their influence on the possible reaction sequences, the diffusion rates of the reactants and products, and the occurrence of either localized pitting or uniform surface recession.

### 2.1 Oxidation of SiC

**Fig. 2:**
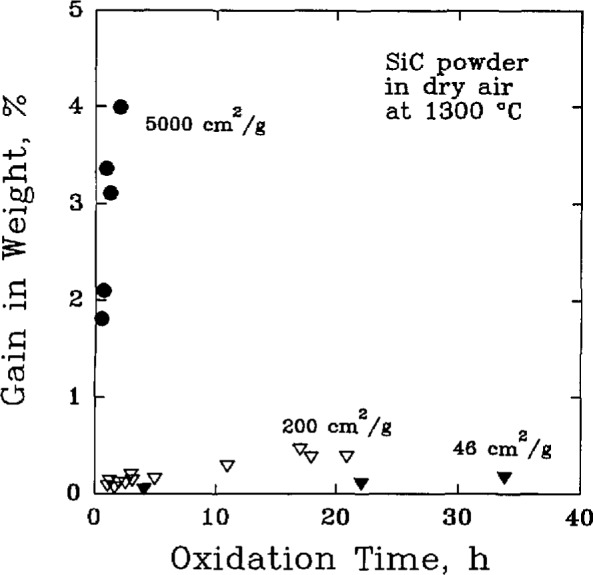
The oxidation of SiC powders, of various specific surface areas, in dry air at 1300 °C. (After G. Ervin, Jr., J. Am. Ceram. Soc. **44** (9), 347–352 (1958).)

The oxidation of SiC has been a subject of considerable interest for many decades [24]. Over this period, the research efforts have shown a progression from studies of the coarser aspects of oxidation to more detailed investigations of contributing mechanisms. It was noted in an early work that the formation of SiO_2_ on the surface, Fig. 2, was probably the rate-controlling process in the normal oxidation of SiC [25]. According to the free energy changes, Table 1, the formation of SiO_2_ should result predominantly from the reaction

$$\text{SiC}+2O_{2}\to {\text{SiO}}_{2}+{\text{CO}}_{2}.$$

In that same work, a weight loss, rather than a weight gain, was observed, Table 2, when SiC was heated in a vacuum. The suggestion was made that the formation of SiO gas was most likely responsible for the latter result, primarily from the reaction

$$\text{SiC}+\left(3/2\right)O_{2}\to \text{SiO}+{\text{CO}}_{2}$$

In a later investigation of the kinetics of SiC oxidation, the oxidation process was resolved into passive and active processes as was done for the oxidation process of silicon [26]. The occurrence of a passive oxidation process, involving weight gain, or an active oxidation process, involving weight loss, Table 3, depended on the magnitude of the oxygen partial pressure [27]. The weight loss was again related to the formation of SiO gas, but the key reaction producing the SiO gas was thought to be

$$\text{SiC}+O_{2}\to \text{SiO}+\text{CO}$$

More generally, for a given temperature, it was found that the oxidation of SiC was passive unless the partial pressure of oxygen was less than a critical value. Alternatively, for a given partial pressure of oxygen, an active-to-passive transition temperature could be defined, Table 4, such that passive oxidation would occur at temperatures less than the transition temperature, while active oxidation would occur at higher temperatures [28].

**Fig. 3:**
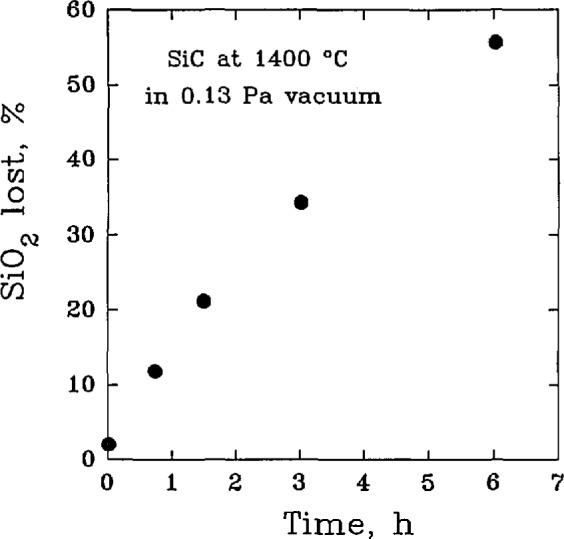
The reaction of silica with silicon carbide in vacuum at 1400 °C. (After W. W. Pultz et al., Trans. Farad. Soe. 62,2499–2504 (1966).)

An additional complication was noted in another high temperature study of kinetics where it was found that the oxide layer itself could react with SiC, Fig. 3, resulting in a loss of SiO_2_ along with the production of SiO gas [29] by the reaction sequence:

$$\begin{array}{l} 2\;\text{SiC}+{\text{SiO}}_{2}\to 3\;\text{Si}+2\;\text{CO},\\ 2\;\text{Si}+\text{CO}\to \text{SiO}+\text{SiC},\\ \text{Si}+{\text{SiO}}_{2}\to 2\;\text{SiO}. \end{array}$$

**Fig. 4:**
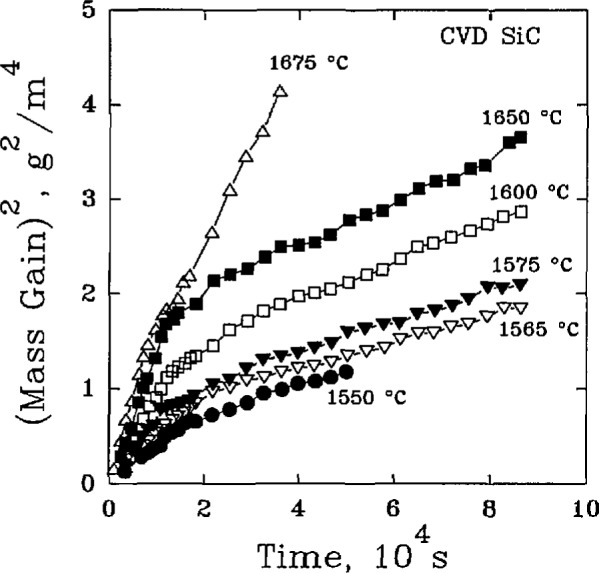
The oxidation of CVD-SiC showing a two-step parabolic oxidation process. (After T. Narushima et al., J. Am. Ceram. Soe. 72 (8), 1386–1390 (1989).)

From these early works, it rapidly became clear that the oxidation mechanisms for SiC were quite complex, and that understanding the mechanisms would require careful examination of the phase equilibria relations among the primary constituents and reaction products [30,31,32]. Further, it was noted that there were large, quantitative differences among the results of kinetics studies that had to be explained, such as the variation of activation energies by an order of magnitude and the occurrence of time-dependent oxidation mechanisms as suggested by Fig. 4. Thus, the roles of materials processing [33,34,35,36], Table 5, and specific molecular transport mechanisms [37,38], had to be considered as integral parts of the analysis of the oxidation of SiC.

**Fig. 5:**
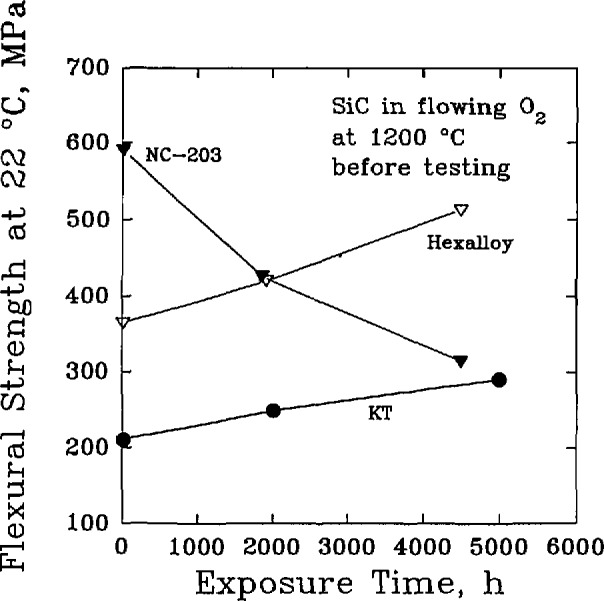
The room-temperature flexural strength of commercial silicon carbide materials after the materials were exposed to flowing oxygen at 1200 °C. (After P. F. Beeher, J. Am. Ceram. Soe. 66 (8), C-120-C-121 (1983).)

Finally, it was recognized that the mechanical performance of a corroded material could be significantly different from the original, uncorrupted material. The principal concern was that the brittle failure behavior of a ceramic could be affected by changes in the surface, or near surface, flaw population. Passive oxidation could, in fact, have a beneficial effect by healing surface cracks and increasing the average flexural strength, while active oxidation could lead to new surface flaws that would decrease the average flexural strength [39]. The roles of material processing and composition were found to be important factors for both the distribution of strength [40], Tables 6 and 7, and the retention of strength, Fig. 5, under long-term exposures to oxidizing environments [41]. Time-dependent effects such as creep and slow crack growth were also noted as important factors in determining high-temperature materials reliability [42].

### 2.2 SiC in Hot Gases

**Fig. 6:**
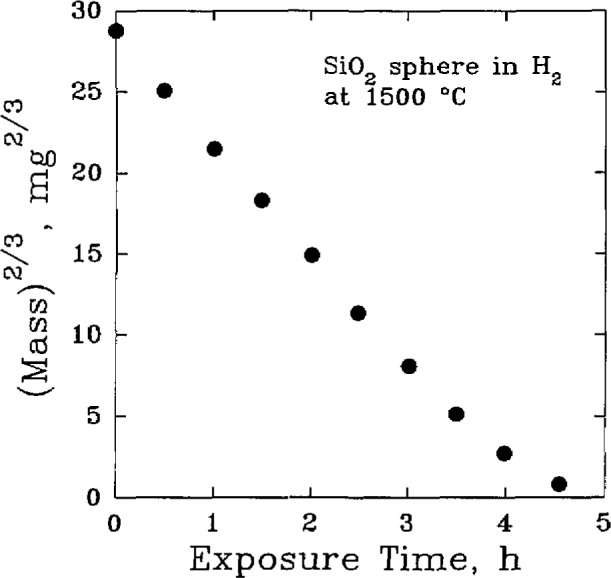
The hydrogen reduction of silica at 1500 °C. (After D. W. Readey, Ceram. Trans. 10, 53–80 (1989).)

The potential applications of silicon carbide include metal and chemical processing industries, coal gasification, and various heat treatment applications that frequently contain H_2_, H_2_O, and N_2_ at temperatures above 1000 °C. Under these gases, the protective silica layer may deteriorate, Fig. 6, thereby exposing the SiC to active oxidation. Under gaseous H_2_, solid silica may be reduced to gaseous products by the reaction [43]:

$${\text{SiO}}_{2}+H_{2}\to \text{SiO}+H_{2}O.$$

In the presence of gaseous H_2_O, the solid SiC may experience active oxidation according to [44]:

$$\begin{array}{l} \text{SiC}+H_{2}O+H_{2}\to \text{SiO}+{\text{CH}}_{4},\\ \text{SiC}+H_{2}O\to \text{SiO}+\text{CO}+2\;H_{2}. \end{array}$$

**Fig. 7:**
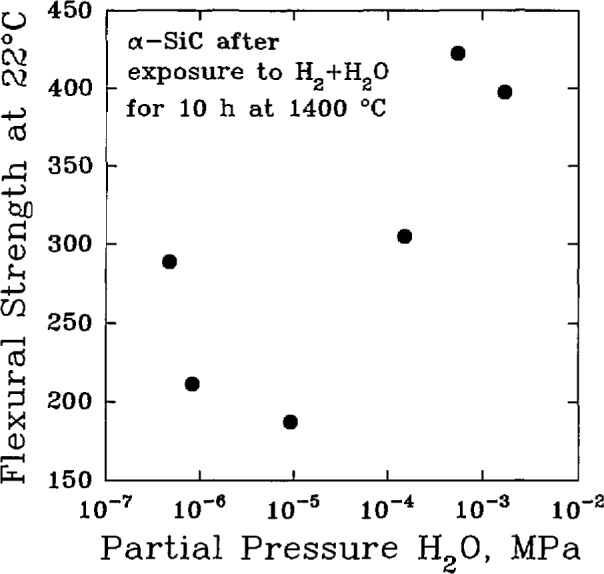
The variation of the room-temperature flexural strength of sintered α-SiC under hydrogen gas with various partial pressures of H_2_O for 10 h at 1400 °C. (After H. Kim et al., Ceram. Trans. 10, 81–96 (1989).)

The primary consequence of such reactions is not the reduced thickness of the component, but rather the increased population of surface flaws that result from the loss of silicon in the form of gaseous SiO. The strength of the material generally decreases as the population of surface flaws increases. Results on the flexural strength of sintered α-SiC, Fig. 7, indicate that when the partial pressure of H_2_O is sufficiently large, the formation of a protective silica layer by the reaction:

$$\text{SiC}+3\;H_{2}O\to {\text{SiO}}_{2}+\text{CO}+3\;H_{2},$$

may become dominant among the various possible reactions between SiC and H_2_O.

**Fig. 8:**
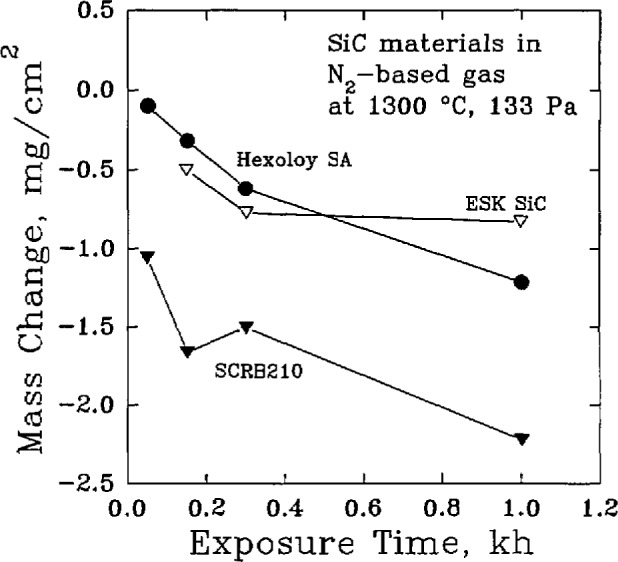
Changes in the mass of commercial silicon carbide materials heated in a nitrogen gas (98.2 vol % N_2_) at 1300 °C, (After D. P. Butt et al., CAM Newslett. 5 (1), 1 (1991).)

Nitrogen-containing environments, Fig. 8, may also result in the active oxidation of SiC [45]. At low gas pressure, SiC may be lost according to:

$$\text{SiC}+\text{CO}+N_{2}+H_{2}\to \text{SiO}+2\;\text{HCN},$$

while at higher pressures, the reaction products may involve solid carbon, silicon nitride, or silicon oxynitride:

$$\begin{array}{l} 2\;\text{SiC}+\text{CO}+N_{2}\to {\text{Si}}_{2}N_{2}O+3\;C,\\ 3\;\text{SiC}+4\;N_{2}\to {\text{Si}}_{3}N_{4}+3\;C,\\ 2\;\text{SiC}+\text{CO}+\left(5/2\right)N_{2}\to {\text{Si}}_{2}N_{2}O+3\;\text{CN}. \end{array}$$

Siliconized SiC may also experience a selective attack against the silicon phase according to:

$$\begin{array}{l} 2\;\text{Si}+\text{CO}\to \text{SiC}+\text{SiO},\\ \text{Si}+\text{CO}+\left(1/2\right)N_{2}+\left(1/2\right)H_{2}\to \text{SiO}+2\;\text{HCN}. \end{array}$$

**Fig. 9:**
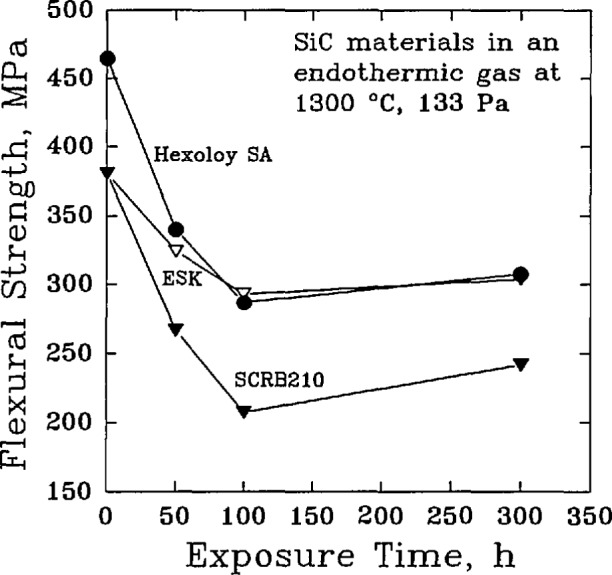
The variation of the room-temperature flexural strength of commercial silicon carbide materials after exposure to an endothermic gas (41.1 vol% H_2_, 37.8 vol% N_2_, 21.1 vol% CO) at 1300 °C. (After D. P. Butt et al., CAM Newslett. 5 (1), 1 (1991).)

In each of these cases, the strength of the material, Fig. 9, can be affected adversely.

### 2.3 SiC in Molten Salts

**Fig. 10:**
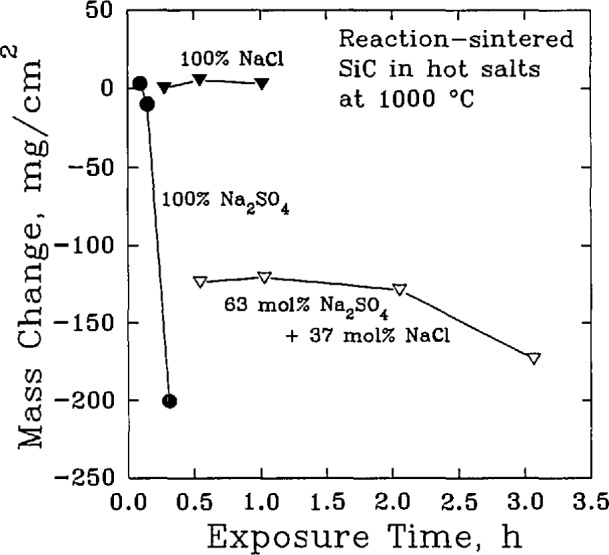
The change in the mass of reaction-sintered silicon carbide exposed to various salts at 1000 °C. (After R. E. Tressler et al., J. Am. Coram. Soc. 59 (5–6) 278–279 (1976).)

Materials used in industrial flue gases, gas turbine environments, aluminum remelting operations, and coal gasification conditions may be exposed to molten salts such as Na_2_SO_4_ and Na_2_CO_3_ which promote the deterioration of the silica layer [46]. The dissolution of the silica layer by Na_2_CO_3_ is thermo-dynamically favorable and may proceed directly according to the reaction

$${\text{Na}}_{2}{\text{CO}}_{3}+{\text{SiO}}_{2}\to {\text{Na}}_{2}{\text{SiO}}_{3}+{\text{CO}}_{2},$$

with a free energy change of −79.9 kJ/mol [47]. In high concentrations, Na_2_SO_4_ can be very corrosive also, Fig. 10, for materials such as reaction sintered SiC. However, the direct reactions of Na_2_SO_4_ and SiO_2_

$$\begin{array}{l} {\text{Na}}_{2}{\text{SO}}_{4}+2\;{\text{SiO}}_{2}\to {\text{Na}}_{2}O\cdot 2\;{\text{SiO}}_{2}+{\text{SO}}_{3},\\ {\text{Na}}_{2}{\text{SO}}_{4}+{\text{SiO}}_{2}\to {\text{Na}}_{2}O\cdot {\text{SiO}}_{2}+{\text{SO}}_{3}, \end{array}$$

occur with free energy changes of +110 kJ/mol and + 135 kJ/mol, respectively, and therefore are not thermodynamically favorable under equilibrium conditions [48]. While the direct reaction of Na_2_SO_4_ and SiC is highly favorable

$$\begin{array}{l} {\text{Na}}_{2}{\text{SO}}_{4}+2\;\text{SiC}+4\;O_{2}\to {\text{Na}}_{2}O\cdot 2\;{\text{SiO}}_{2}+{\text{SO}}_{3}+2\;{\text{CO}}_{2},\\ {\text{Na}}_{2}{\text{SO}}_{4}+\text{SiC}+2\;O_{2}\to {\text{Na}}_{2}O\cdot {\text{SiO}}_{2}+{\text{SO}}_{3}+{\text{CO}}_{2}, \end{array}$$

with free energy change −1855 kJ/mol and —845 kJ/mol, respectively, the silica layer prevents this direct reaction [49]. Instead, two indirect reaction schemes by which Na_2_SO_4_ may corrode SiC are considered more probable. The first scheme involves reaction with carbon, either from the aggressive environment or from excess free carbon in the SiC material, to form basic Na_2_O or Na_2_S according to

$$\begin{array}{l} {\text{Na}}_{2}{\text{SO}}_{4}+C\to {\text{Na}}_{2}O+{\text{SO}}_{2}+\text{CO},\\ {\text{Na}}_{2}{\text{SO}}_{4}+2\;C\to {\text{Na}}_{2}S+2\;{\text{CO}}_{2}. \end{array}$$

These compounds then attack the protective silica layer according to

$$\begin{array}{l} {\text{Na}}_{2}O+\text{Si}O_{2}\to {\text{Na}}_{2}{\text{SiO}}_{3},\\ {\text{Na}}_{2}S+\text{Si}O_{2}+\left(3/2\right)O_{2}\to {\text{Na}}_{2}{\text{SiO}}_{3}+{\text{SO}}_{2}, \end{array}$$

and, hence, lead to the active corrosion, Table 8, of the SiC material [50].

In the second scheme, liquid Na_2_SO_4_ dissociates into solid Na_2_O and gaseous SO_3_, and the Na_2_O subsequently interacts with the silica to form a liquid sodium silicate [51]:

$$\begin{array}{l} {\text{Na}}_{2}{\text{SO}}_{4}\to {\text{Na}}_{2}O+{\text{SO}}_{3},\\ 2\;{\text{SiO}}_{2}+{\text{Na}}_{2}O\to \left({\text{Na}}_{2}O\right)\cdot 2\left({\text{SiO}}_{2}\right). \end{array}$$

It is also possible for a hybrid of these two schemes to occur to form a self-sustaining fluxing mechanism when the hot gaseous environment contains a small partial pressure of SO_3_. In this case, the Na_2_SO_4_ reacts with the free carbon in the substrate to produce Na_2_O or Na_2_S which subsequently reacts with the silica layer to form Na_2_SiO_3_. The latter product may subsequently react with the SO_3_ from the atmosphere to produce Na_2_SO_4_.

$${\text{Na}}_{2}{\text{SiO}}_{3}+{\text{SO}}_{3}\to {\text{SiO}}_{2}+{\text{Na}}_{2}{\text{SO}}_{4}.$$

**Fig. 11:**
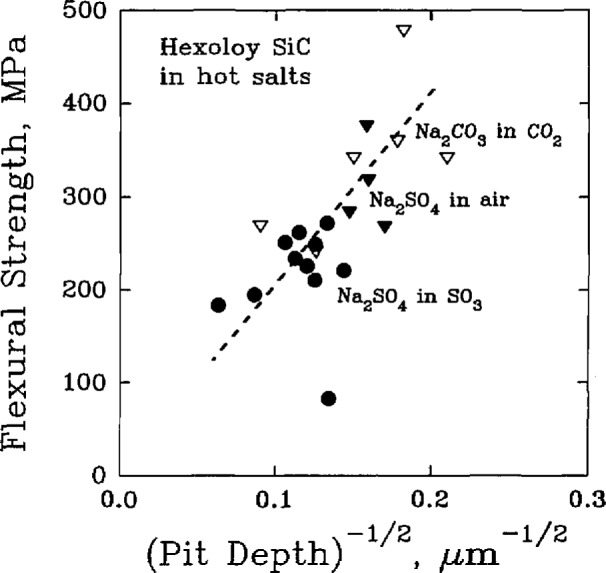
The correlation of flexural strength with the corrosion pit depth for a commercial silicon carbide material exposed to various hot salts. (After J. L. Smialek et al., J. Am. Ceram. Soc. 69 (10), 741–752 (1986).)

In each scheme, the corrosive pitting of the SiC material results in a degradation of the flexural strength, Table 9, of the material to an extent that depends, Fig. 11, on the severity of the pitting [52].

### 2.4 SiC in Molten Metals

Corrosion of ceramics by molten metals is not as well described in the literature as corrosion in other environments, although corrosion by molten metals is a concern in the design of heat exchangers for use in aluminum remelt industries, steel reheating furnaces and soaking pits, and other flue gas environments containing metallic species. The primary ceramic being considered for these applications is SiC because of its high thermal conductivity and thermal shock resistance and its potential resistance to corrosion by the molten metals.

The metallic species in the melt principally attack the oxide layer in reactions such as [53]

$$4{\left[\text{Al}\right]}_{\text{melt}}+3\;{\text{SiO}}_{2}\to 2\;{\text{Al}}_{2}O_{3}+3{\left[\text{Si}\right]}_{\text{melt}},$$

thereby exposing the SiC to direct corrosion by gaseous halide or other species in the combustion environment and possibly by metal-containing compounds as in the reaction [54]

$${\text{Fe}}_{2}O_{3}+3\;\text{SiC}\to {\text{Fe}}_{2}{\text{Si}}_{3}+3\;\text{CO}.$$

Sintered SiC, recrystallized SiC, and siliconized SiC were found to have active corrosion rates, Tables 10 and 11, in aluminum remelt and steel soaking pit furnaces, while passive deposits were formed in a steel forge furnace. The mechanical strengths, Table 12, were generally degraded as a result of the increased surface flaws [55].

### 2.5 SiC in Complex Environments

Heat exchangers in coal combustion or coal gasification environments may be exposed to a combination of hot gases, Table 13, and molten slags, Table 14, simultaneously. Such complex environments increase the number of reaction paths that may be involved in the degradation of the protective silica layer and the active corrosion of the substrate. As should be expected, the experimental results produced in such environments are difficult to interpret precisely and may result in apparent contradictions. For example, compared to silicon carbide, alumina has been found to be both less resistant [56] and more resistant [57] to corrosion in slags nominally characterized as acidic.^1^ The significance of this apparent contradiction is not that the results differ, but that a more precise characterization of the envirionment and the materials is needed along with consideration of the possible transport phenomena, such as fluxing, that can affect the overall nature of the corrosive process. In general, it can be noted that the corrosion of structural ceramics in complex environments tends to result in a net loss of material, Table 15, and the extent of the loss varies with the relative amount of basic and acidic components in the slag [58,59]. Acidic slags typically produce recession rates on the order of 1 mm/yr for silicon carbide at high temperature, while the recession rates from basic slags are on the order of 10–100 mm/yr.

Part of the dependence on the acidity or basicity of the slag may result from the order of magnitude difference in the viscosities of these slags at high operating temperature. The viscosity of an acidic slag is estimated to be approximately 60 Pa · s (600 poise), while being only 5 Pa · s (50 poise) for a basic slag [60]. The lower viscosity of the basic slag should be more conducive to the diffusion of the reactant and product species and thus to a more extensive material loss.

Specific chemical contributions to the differences between acidic and basic slags are thought to result from the greater presence of iron and calcium compounds in the basic slags. CaO may interact with the silica layer to form calcium silicate compounds, thereby reducing the effectiveness of the silica layer [61]. Localized, active corrosion of the SiC may occur when the iron compounds react directly with the SiC subsrate to form iron silicides with an effective reaction of the form [62]:

$$13\;\text{SiC}+5\;\text{Fe}\left(\text{slag}\;\text{matrix}\right)\to {\text{Fe}}_{5}{\text{Si}}_{13}+13\;C.$$

This localized corrosion mechanism is thought to be important particularly for a slag thickness of 100 μm or greater and a temperature of 1250 °C or higher. Under these conditions, bubble formations from the evolution of gaseous SiO and CO may disrupt the silica layer, thereby providing the means for a direct reaction between the slag and the substrate.

Corrosion affects the lifetime of a material under in-service conditions not only through the loss of material, but also through the variation of the strength of the material. Corrosion increases the population of surface flaws which degrades the strength of the material and reduces its lifetime by increasing the probability of fracture under a mechanical or thermally induced stress. The strengths of SiC materials at elevated temperature were measured by C-ring tests, Table 16 [63], and four-point bend tests, Table 17 [64], after exposing the materials to coal slags at various temperatures for various amounts of time. From these results, it may be readily inferred that the degradation of the strength of the material due to a temperature rise is compounded by oxidation, and both of these effects are significantly compounded by reaction with the slag. It is also significant that corrosion pits were cited as the primary failure sites of the exposed samples in both the C-ring tests and the four-point bend tests.

## 3. Si_3_N_4_

Silicon nitride exists in two crystallographic phases, denoted α-Si_3_N_4_ and β-Si_3_N_4_ [65]. The α-Si_3_N_4_ phase transforms to the β-Si_3_N_4_ form at approximately 1500 °C. Silicon nitride powders may be prepared by several different methods. The direct nitridation of silicon powder produces silicon nitride by the reaction

$$3\;\text{Si}+2\;N_{2}\to {\text{Si}}_{3}N_{4}.$$

The carbothermic reduction of silica may also be used according to

$$3\;{\text{SiO}}_{2}+6\;C+2\;N_{2}\to {\text{Si}}_{3}N_{4}+6\;\text{CO}.$$

Amorphous silicon nitride with very fine particle sizes can be produced by vapor phase methods using reactions such as

$$\begin{array}{l} 3\;{\text{SiCl}}_{4}+4\;{\text{NH}}_{3}\to {\text{Si}}_{3}N_{4}+12\;\text{HCl},\\ 3\;{\text{SiH}}_{4}+4\;{\text{NH}}_{3}\to {\text{Si}}_{3}N_{4}+6\;H_{2}. \end{array}$$

Silicon nitride powders can be processed by a variety of methods to produce bulk materials for structural applications. Common methods include hot pressing, sintering, hot isostatic pressing, and sintering of reaction-bonded compacts [66]. Sintering aids such as MgO and Y_2_O_3_ are usually used with all of the methods to produce high density products. In hot pressing, uniaxial stresses on the order of 15–30 MPa may be applied to the powder for several hours at temperatures in the range 1650 to 1850 °C. Pressureless sintering, in contrast, is conducted in a nitrogen atmosphere of approximately 0.1 MPa in the temperature range 1600 to 1800 °C. In both cases, boron nitride may be used for various purposes in containing or manipulating the product material with the result that the surface of the product may have a small boron nitride contamination. The final product is generally a polycrystalline material consisting primarily of elongated, fiber-like, *β*-Si_3_N_4_ grains and a secondary, intergranular phase that may be either glassy MgO or crystalline Y_2_O_3_. When MgO is used as an additive in quantities greater than 5 wt. %, crystalline Mg_2_SiO_4_ may also be present. For reaction-bonded silicon nitride fired in a lower temperature range, 1150 to 1400 °C, the final product may consist predominantly of *α*-Si_3_N_4_ grains with a lower bulk density and a significantly larger porosity, on the order of 10%–30%.

### 3.1 Oxidation of Si_3_N_4_

The oxidation of silicon nitride is a complex process that depends significantly on the sintering aids and the impurities in the material [67]. The primary protective scale is silica which may be formed directly from the reaction of silicon nitride and oxygen:

$${\text{Si}}_{3}N_{4}+3\;O_{2}\to 3\;{\text{SiO}}_{2}+2\;N_{2}.$$

This reaction may be most significant during the initial oxidation of the substrate. Subsequently, multistep reaction sequences may be more important.

In the case of chemical vapor deposited (CVD) silicon nitride without sintering aids [68], the formation of a duplex oxide scale has been reported. The scale was found to consist of an outer layer of silica and an inner layer of silicon oxynitride. The oxidation process in this case could be described by two reaction steps. First, oxygen diffused through the silica layer to the silicon oxynitride layer where part of the oxygen reacted to form additional silica according to:

$${\text{Si}}_{2}N_{2}O+\left(3/2\right)\;O_{2}\to 2\;{\text{SiO}}_{2}+N_{2}.$$

The unreacted oxygen continued to diffuse to the substrate material where additional silicon oxynitride was formed:

$$2\;{\text{Si}}_{3}N_{4}+(3/2)O_{2}\to 3\;{\text{Si}}_{2}N_{2}{O+N}_{2}.$$

Diffusion through the silicon oxynitride layer was identified as the rate-controlling process because the molecular oxygen diffusivity, Table 18, was significantly smaller for Si_2_N_2_O than for SiO_2_. The overall oxidation process, though, followed parabolic kinetics,

$$x^{2}=2k_{p}t,$$

in which the square of the scale thickness is proportional to the time of exposure, with a parabolic rate constant, *k*_p_, of 66 nm^2^/min.

**Fig. 12:**
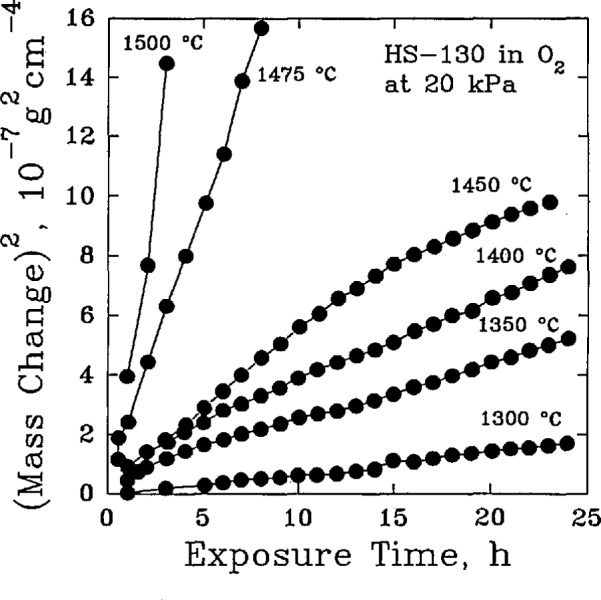
The oxidation of a commercial silicon nitride material in dry oxygen showing parabolic oxidation kinetics. (After W. C. Trip et. al., J. Am. Ceram. Soc. 59 (9–10), 399–403 (1976).

When sintering aids or impurities have been present, the composition of the scale has been found to be more complex. For hot-pressed Si_3_N_4_ containing MgO and CaO as additives, the scale consisted of glassy SiO_2_, in which crystalline α-cristobalite also formed, plus magnesium silicates and magnesium-calcium silicates [69]. A similar study analyzed the composition of the scale, Table 19, and found that MgSiO_3_ could be clearly identified as a principal component of the scale [70]. Other compounds, while evidently present, could not be identified conclusively. The oxidation kinetics relation was parabolic, Fig. 12, as has been found to be common for most MgO-containing silicon nitrides [71], with an activation energy of approximately 440 kJ/mol [72].

The oxide scale formed on sintered silicon nitride containing yttria as a major sintering aid has been found to differ both substantially and morphologically from the scale on Si_3_N_4_ prepared either by hot pressing with MgO or by the CVD technique [73]. The scale itself was found to be a layered structure in which each layer had an outer region of crystalline silica, an intermediate glassy region, and an inner thin layer of Y_2_Si_2_O_7_. The glassy region was porous, often containing large bubble formations that most likely resulted from the evolution of N_2_ gas during oxynitride formation. Thin layers of Y_2_Si_2_O_7_ were also found at the interface between the substrate and the scale, and at the outer surface of the scale.

It was suggested that the morphology of the scale had a direct effect on the apparent oxidation kinetics of the material. Initially, the scale growth was logarithmic rather than parabolic, such that

$$x=k_{1}+k_{2}\;\ln(t),$$

for constants *k*_1_ and *k*_2_. It was reasoned that such a relation would be appropriate if the effective cross section through which diffusion could occur diminished with time, as might have occurred when gas bubbles grew in the scale. Subsequently, when a sufficient cristobalite layer had formed, the slow diffusion rate through that layer would be rate-controlling, and the kinetic relation would become parabolic. A behavior consistent with this scheme was also observed in a study using silicon nitride sintered with Y_2_O_3_ and Fe_2_O_3_ [74]. In that work, the kinetics relation was described as being asymptotically parabolic with an apparent activation energy of 470 kJ/mol.

At sufficiently low oxygen partial pressure, the passive oxidation of silicon nitride may change to active oxidation [75]. The temperature at which this transition occurs for a given oxygen pressure, Table 20, increases with increasing oxygen pressure [76].

The strength of both sintered and hot pressed silicon nitride materials have been found to decrease for oxidation times of one hour or longer [77]. A brief increase in strength was noted for very short oxidation times, approximately 30 min, which was thought to be due to the blunting of crack tips. For longer times, Table 21, the oxidation of the material resulted in the generation of new flaws that caused the average strength to decrease. Oxidizing the two materials under static load, however, resulted in distinct behaviors. The sintered material had an increase in strength, while the hot-pressed material suffered a decrease in strength.

### 3.2 Si_3_N_4_ in Hot Gases

The passive oxidation of hot-pressed silicon nitride has been found to be enhanced by water vapor in oxygen atmospheres [78], with the parabolic rate constant, Table 22, being larger than for dry oxygen. The activation energy for oxidation was 488 ± 30 kJ/mol in wet oxygen and 375 ± 25 kJ/mol in dry oxygen.

The presence of gaseous H_2_O in H_2_ gaseous environments has been found to cause an active oxidation of Si_3_N_4_ according to [79]:

$${\text{Si}}_{3}N_{4}+3\;H_{2}O\to 3\;\text{SiO}+2\;N_{2}+3\;H_{2}.$$

Under these conditions, the average flexural strength of the material, Table 23, decreased. When the partial pressure of H_2_O was greater than about 10 Pa, a protective silicate layer may have formed as a result of a reaction between the gaseous reaction product, SiO, and the sintering aid, Y_2_O_3_. Concurrently, the flexural strength of the material increased.

### 3.3 Si_3_N_4_ in Molten Salts

The corrosion of silicon nitride by molten salts has been found to occur in three stages. For sodium carbonate on reaction-bonded Si_3_N_4_, the first stage is a very brief process, lasting only minutes, during which sodium silicate is formed. The second stage follows diffusion-limited parabolic reaction kinetics resulting from the inward diffusion of molecular oxygen to the silicon nitride surface where the subsequent reaction forms silica. The silica material, though, partially dissolves into the silicate glass until a saturation limit is reached. Thereafter, oxidation produces a growing silica layer. Oxygen diffusion through this layer is very slow, and becomes the rate limiting step for the third stage of the corrosion process [80].

A similar study on hot isostatically pressed reaction-bonded Si_3_N_4_ obtained very similar results [81]. The brief first stage was found to be a period of weight loss controlled by two reactions. In the first, gaseous oxygen reacted with the silicon nitride to form a solid silica layer according to:

$${\text{Si}}_{3}N_{4}+3\;O_{2}\to 3\;{\text{SiO}}_{2}+2\;N_{2}.$$

Subsequently, the silica dissolved in the molten salt layer to form a liquid sodium silicate:

$$x\;{\text{SiO}}_{2}+{\text{Na}}_{2}{\text{CO}}_{3}\to {\text{Na}}_{2}O\cdot x({\text{SiO}}_{2})+{\text{CO}}_{2}.$$

The first stage process was completed after approximately 5 min after which weight gain occurred primarily due to the formation of silica.

Corrosion of silicon nitride by sodium sulfate was found to have a similar corrosion process consisting, however, of only two stages, an initial lengthy period of slow weight loss followed by a process of slow weight gain. The weight loss appeared to be mostly the result of the vaporization of the Na_2_SO_4_. The primary reaction sequence for corrosion was a coupled oxidation-dissolution process such that:

$$\begin{array}{l} {\text{Si}}_{3}N_{4}+3\;O_{2}\to 3\;{\text{SiO}}_{2}+2\;N_{2},\\ x\;{\text{SiO}}_{2}+{\text{Na}}_{2}{\text{SO}}_{4}\to {\text{Na}}_{2}O\cdot x({\text{SiO}}_{2})+\\ {\text{SO}}_{2}+(1/2)\;O_{2}. \end{array}$$

Silica formation and dissolution proceeded with the silica layer growing at a rate that was faster than what was expected for normal oxidation. Eventually, the silica layer became sufficiently thick that further oxidation of the silicon nitride substrate was limited by the slow diffusion of oxygen through the silica layer.

### 3.4 Si_3_N_4_ in Molten Metals

Hot pressed silicon nitride materials, with either Y_2_O_3_ or MgO as sintering aids, have been exposed to the flue gas of an aluminum remelting furnace in which the flue gas consisted mainly of CO_2_, CO, O_2_, and H_2_O, plus small amounts of Cl, F, SO_2_, SO_3_, NO, and NO_2_. After exposure, the silicon nitride specimens were found to be coated with a vitreous deposit in which β-Si_3_N_4_, Si, and Al_2_O_3_ were found [83]. The Si_3_N_4_ material apparently diffused or dissolved from the substrate and was absorbed into the deposit, while the Si and A1_2_O_3_ were derived from the aluminum melt. The deposit also showed evidence of trapped gas bubbles, and some depletion of the sintering aid components was found in the surface region of the substrate material adjacent to the vitreous deposit. The latter result indicated that it was primarily the inter-granular phases that were corroded. Specimens with MgO as the sintering aid appearred to be less resistent to corrosion than specimens with Y_2_O_3_ as the sintering aid, as was reflected in the variations of the flexural strengths of the materials, Table 24. While both materials had significantly reduced flexural strengths, the residual strength of Si_3_N_4_(Y_2_O_3_) was nearly twice that of Si_3_N_4_(MgO).

### 3.5 Si_3_N_4_ in Complex Environments

An extensive penetration of coal slag into hot pressed NCX-34 silicon nitride was found as a result of corrosion in simulated coal combustion environments (slags 5 and 6 in Table 14) [84]. This material had WC and β-Y_5_Si_3_O_12_N as minor phases in the material. It was noted that the oxidation of Y_5_Si_3_O_12_N to form Y_2_SiO_5_, cristobalite SiO_2_, or RY_5_Si_6_O_2_, where R may be H, Na, Mn, Fe, Al, Th, or Zr, would occur with a large increase in molar volume. The resulting tensile stress could cause microcracking and thus would promote slag penetration and the formation of subsurface pits. These microcracks and pits were cited as being responsible for the strength degradation, Table 25, observed in these environments. When the corroded layer of the surface was removed by diamond grinding, the strength of the material returned to approximately 95% of its original, as-received value. Thus the strengh-reducing defects were limited to the surface of the specimens and did not involve internal compositional changes in the substrate.

## 4. Coating Systems

The primary corrosion resistance of both silicon carbide and silicon nitride results from the oxide layer formed on the surface of the material. It is reasonable, therefore, to consider the possibility of enhancing the corrosion resistance of these materials by modifying their surfaces with specially prepared oxide coatings. For such coatings, there would be chemical, thermal, and mechanical requirements beyond the need to resist corrosion by combustion gases, slag, and ash. The coating and substrate materials would need to be well bonded but otherwise would have a low rate of subsequent reaction, and their coefficients of thermal expansion would need to be compatible to prevent thermally induced stresses and subsequent cracking. The coating also would need to have adequate toughness and thermal shock resistance, as well as a sufficiently high thermal conductivity, so that the performance of the system would not be affected adversely.

Initial insight into the selection of candidate coating materials may be derived from a consideration of the phase equilibria relations between the slag and ash constituents and the candidate oxide coatings. For slagging conditions in particular, the major slag constituents are silica, alumina, and iron oxide; hence, coatings of these same oxides would be expected to be dissolved by the slag. Other slag constituents such as alkali and rare earth oxides could also react with these coatings to produce lower melting point compositions which would degrade the performance of the coatings. Consequently, silica, alumina, and iron oxide would not be expected to be well suited as protective coatings in these coal-fueled applications.

Little work appears to have been reported on the use of ceramic coatings on ceramics. However, preliminary indications of the potential for such coatings may be gained from current studies of refractory oxide coatings and from ceramic coatings on metal alloys in turbine engines.

Refractory oxide coatings have been used successfully in the radiant heater sections of pulverized coal-fueled boilers to protect low-alloy water walls. In these slagging applications, a layer of solidified slag underlies the molten deposit and limits the solution of the refractory coating into the slag deposit. General summaries of research on the behavior of refractory oxides in coal gasification systems indicate that alumina refractories [85] might be suitable for use in dry ash systems to 1100 °C and that magnesia-chromia refractories [86] might be useful in slagging systems at temperatures of 1500 °C. However, it should be noted that these applications exhibit lower oxygen partial pressures and higher water partial pressures than would be expected in combustion systems, and the refractories examined in these studies were in brick or castable forms with significant amounts of other phases present.

There is a significant body of research on the stability of oxide-forming elements in alloys in coal gasification and combustion applications. Much of this research has been conducted with a focus on the propensity for sulfidation [87]. The results have clarified the regimes of oxygen, sulfur [88], and chlorine [89] partial pressures in which protective oxides are stable and have helped to understand alloy corrosion mechanisms [90].

Table 26 shows the lower partial pressure limits of stability for several oxides in oxygen and sulfur, The values shown in the table are many orders of magnitude lower than any value that might be expected to be encountered in combustion systems. As a result, none of these oxides would have an intrinsic problem in terms of stability.

Among the materials listed in Table 26, the relatively high coefficient of thermal expansion of zirconia has encouraged its use as a thermal barrier coating on metallic gas turbine components. This application has required investigation of the corrosion of zirconia-based coatings in combustion environments. Typically, low velocity burner rig tests have been conducted on compositionally graded coatings [91]. In these experiments, corrosive alkali sulfates and sulfates with vanadium were deposited on zirconia containing 8% yttria as a stabilizing oxide. No significant reaction of zirconia with sodium sulfate was noted. However, the addition of vanadium and magnesium, at the level of 50 parts per million, was shown to foster corrosive reactions and the destabilization of the zirconia. Significantly, the porosity of the coating also was shown to foster coating failures due to the penetration of liquid sodium sulfate into the coating [92]. Under thermal cycling, the liquid sodium sulfate was thought to have frozen and expanded, thereby mechanically opening cracks in the coating. Failure in these experiments, Table 27, was determined as the onset of cracking or spallation. Coating lifetimes greater than 500 h were reported.

Concern over the role of vanadium in the deterioration of stabilized zirconia coatings has resulted in research on the reactivity of indium oxide, scandium oxide, yttria, and magnesia stabilizers. Thermogravimetric analyses of these oxides in molten sodium vanidate equilibrated in an SO_3_ environment has shown indium oxide and scandium oxide to be more resistant to attack than the conventional stabilizers, yttria and magnesia [93].

Finally, attempts are being made to improve the compatibility of ceramic coatings and metal substrates. The effort is specifically designed to reduce the thermal stresses caused by the different amounts of thermal expansion experienced by metals and ceramics. The approach being taken is to use intermediate layers of materials to provide a gradual transition in the coefficient of thermal expansion across the interface region [94]. For metallic substrates, the intermediate layers typically have consisted of mixed ceramic and metallic compositions which unfortunately provides an opportunity for corrosion to occur beneath the ceramic over-layer. At the present time, therefore, the use of ceramic coatings metallic interlayers does not appear to offer a significant advantage for coal-fueled applications.

## 5. Conclusion

Significant progress towards a comprehensive understanding of the oxidation of both silicon carbide and silicon nitride has been made over the past several decades. Models have been advanced to account for the quantitative effects of competing chemical reactions, required mass transport mechanisms, and changes in surface and microstructural morphologies. In contrast, models describing the corrosion of these materials in complex environments are considerably more speculative. Few studies have been made to isolate the effects of specific components of either the complex atmosphere or the material. Most recent studies involving complex environments have been focused on the more immediate concerns of engineering design, viz., does the given material have adequate corrosion resistance for the given design objective? Important clues towards the development of better materials can be provided by such studies, particularly when analyses of the compositions of the starting materials and environments are given along with the compositions of the reaction products. However, the development of detailed models must rely on known results of specific interactions. Consequently, much of the understanding of corrosion in complex environments is based on detailed studies of oxidation or of corrosion in simplified environments.

The silica layer, for example, that develops at the surface of the SiC or Si_3_N_4_ substrate, is known to play a key, multifaceted role in the corrosion resistance of both materials. The frequently observed parabolic corrosion kinetics may be directly related to the diffusion of oxygen through an oxide layer, but there are numerous reaction sequences that can contribute to that result.

For silicon carbide, silica forms a protective barrier that prevents the direct reaction of the substrate with an attacking species. The diffusion of oxygen through the silica results in parabolic reaction kinetics. The oxide barrier, however, may be sacrificial in the sense that the silica itself may be dissolved in a molten deposit. Replenishment of the silica layer then depends on the diffusion of oxygen to the substrate where additional silica can be formed by the oxidation of the substrate. If the attacking deposit or the silica layer impedes the flow of oxygen to the substrate, such that the partial pressure of oxygen is very low, then the silica itself may react directly with the substrate causing both reactants to be diminished.

For silicon nitride, the oxidation of the substrate forms silicon oxynitride which subsequently oxidizes to form silica. In this case, parabolic reaction kinetics are still observed, but the layer of silicon oxynitride may control the oxygen diffusion rate.

The dominant reaction sequence at any time may be strongly influenced by the chemical species in the attacking atmosphere and by the impurities, sintering aids, grain boundary phases, and porosity of the substrate material, as well as the immediate reaction history. Impurities such as carbon, for example, may cause an acidic slag to behave more like a basic slag with respect to corrosion of silicon carbide. For silicon nitride, sintering aids such as MgO and CaO may add magnesium-calcium silicate components to the oxide layer and may influence the crystalline or glassy nature of the oxide. Additional quantitative sudies are needed to investigate these effects in greater detail.

In the context of designing components for use in corrosive environments, there are two primary concerns regarding the selection of a material: the survival of the material, expressed as the recession rate of the surface, and the mechanical strength of the component. The corrosion rates for ceramics can be relatively small, and, as a result, the recession rates may be tolerable. Recession rates of SiC on the order of 1 mm/yr in acidic slag and 10–100 mm/yr in basic slags are possible. However, surface pitting and the overall increase in the surface flaw populations generally degrade the strength of the material and can reduce its average mechanical lifetime even when the recession rate is small. While some combinations of material and environment may produce a short-term strength enhancement resulting from the healing of surface defects, long-term corrosion generally tends to decrease the strength of the material as the severity of the surface pitting increases. An understanding of the interplay of the time-dependent chemical and mechanical effects and consequences has yet to be established.

There are several critical issues that have yet to be examined. Prominent among these issues is long-term corrosion. Most studies report short-term results that span only a few tens of hours, and results with exposure times on the order of 1000 h are scarce. Long-term data are particularly needed to study possible time-dependent phenomena such as break-away corrosion and the potential for lifetime-limiting effects such as environmentally enhanced creep phenomena.

Of equal importance is the need for systematic studies of corrosion in environments that are well characterized chemically and thermally. Studies conducted in industrial furnaces are valuable proof tests, but the results are often difficult to interpret because the precise composition of the environment is unknown and both the composition and the temperature distribution may vary over the course of the study.

In conjunction with these tests, mechanical properties need to be measured in situ at elevated temperature. Secondary or grain boundary constituents, impurities, and reaction products can be liquids at the temperatures of interest. Measurements at room temperature do not properly reflect the consequences of these phases, nor do they account for environmentally assisted fracture effects.

Lastly, while it has been noted that the corrosion behavior of various types of silicon carbide and silicon nitride have been examined in many environments, little research has focused on the role of ceramic coatings in protecting silicon-based materials. It may be possible to enhance the corrosion resistance of silicon-based ceramics by the use specially designed oxide coatings. Coatings of either the overlay type, which may be applied by plasma spraying, or surface modified materials offer the potential to develop surface layers more resistant to reaction with alkali or slag deposits than native silica. Consideration of previous experience with coatings for refractory ceramics and metal alloys suggests that zirconia stabilized with indium oxide or scandium oxide might provide an effective coating for silicon-based ceramics. However, the extent of corrosion may depend on the specific slag and ash deposit compositions, and the degree of protection may depend significantly on the capacity of the coating to resist spallation and cracking. Measurements of the thermal and mechanical properties of such coatings, along with studies of potential problems associated with the porosity of the coating, would have to be made.

## Figures and Tables

**Table 1:** Reactions of SiC with O_2_ and associated free energy changes, Δ*F*^a^

	Δ*F* (kJ/mol)	
	25 °C	1627 °C
SiC + 2 O_2_→SiO_2_ + CO_2_	−1168.0	−901.2
SiC+ (3/2) O_2_→SiO_2_ + CO	− 910.9	−783.2
SiC+O_2_→SiO_2_ + C	− 773.6	−505.0
SiC+(3/2)O_2_→SiO + CO_2_	− 460.7	−604.6
SiC + O_2_→SiO + CO	− 203.3	−486.6
SiC+(l/2)O_2_→SiO + C	− 66.1	−208.4
SiC + O_2_→Si + CO_2_	− 342.7	−361.9
SiC+(1/2) O_2_→Si + CO	− 85.4	−243.9

a After G. Ervin, Jr., J. Am. Ceram. Soc. **44** (9), 347–352 (1958).

**Table 2:** The oxidation of high purity, 100–200 mesh SiC at low pressure^a^

Air pressure (Pa)	Temperature (°C)	Exposure time (h)	Mass ehange (%)
0.001	1600	16	− 4.2
0.133	1600	17	−19.9
13.332	1440	4	− 2.5

a After G. Ervin, Jr., J. Am. Ceram. Soc. **44** (9), 347–352 (1958).

**Table 3:** Oxidation rate of high purity SiC^a^

Pressure (*P*a)	Temperature (°C)	O_2_ Flow rate (10^17^ molecules per second)	Mass gain (μg/s)
1.2	1150	1.02	0.04
1.2	1200	1.32	0.05
1.2	1200	0.64	0.02
1.2	1250	1.10	−0.19
1.2	1300	1.13	−1.56
1.2	1350	1.04	−1.65
1.2	1400	0.62	−1.31
5.3	1150	8.4	0.52
5.3	1200	7.4	0.06
5.3	1250	10.5	0.97
5.3	1300	8.7	−0.75
5.3	1350	9.3	−0.86
5.3	1400	8.1	−5.22
13.3	1150	52.5	3.6
13.3	1200	56.5	0.2
13.3	1250	56.5	0.8
13.3	1300	56.5	0.2
13.3	1350	64.0	3.9
13.3	1400	66.5	−9.3
66.7	1150	6.3	0.2
66.7	1250	8.0	1.1
66.7	1300	7.6	0.6
66.7	1350	8.4	4.8

a After E. A. Gulbransen et al., J. Elcetrochem. Soe. **113**(12), 1311–1314 (1966).

**Table 4:** Aetive-to-passive transition temperature, *T*_ap_, for sintered α-SiC at various values of the partial pressure of oxygen, P(O_2_)^a^

Flow rate (cm^3^/s)	P(O_2_) (Pa)	*T*_ap_ (°C)
1.66	18.2	1403
1.66	18.8	1405
1.66	65.8	1532
1.66	125.1	1542
0.83	10.2	1389
0.83	55.6	1496
0.83	123.2	1543
0.56	7.3	1373
0.56	16.8	1392
0.56	61.6	1507
0.17	2.5	1347
0.17	17.6	1372
0.17	57.4	1468
0.17	115.9	1532

a After W. L. Vaughn et al., J. Am. Ceram. Soe. 73 (6), 1540–1543 (1990).

**Table 5:** Parabolic rate parameters, *K*_par_, and activation energies, *E*_act_, for the oxidation of SiC in dry oxygen^a^. SCSC = single crystal SiC; BL = black; F = fast growth face; SL = slow growth face; GR = green; CNTD = controlled nucleation thermal deposition SiC; SASC = sintered α-SiC; HPSC = hot pressed SiC

Material	Temperature (°C)	*K*_par_ (nm^2^/min)	*E*_act_ (kJ/mol)
SCSC BL F	1200	712±58	
	1300	620±36	
	1400	1040±76	
	1450	1920±61	
	1500	2310±395	
	1200–1500		134–197
SCSC BL SL	1200	19±5	
	1300	175±91	
	1400	848±228	
	1450	1640±412	
	1500	1770±278	
	1200–1500		372
SCSC GR F	1200	346±47	
	1300	658±38	
	1400	1150±160	
	1450	2310±1060	
	1500	3840±1040	
	1200–1500		121–297
SCSC GR SL	1200	41±6	
	1300	331±72	
	1400	943±179	
	1450	2860±784	
	1500	4970±793	
	1200–1500		339
CNTD	1200	344±53	
	1300	745±203	
	1400	1370±255	
	1450	2690±537	
	1500	4510±758	
	1200–1500		142–293
SASC	1200	175±87	
	1300	540±71	
	1400	949±272	
	1450	1770	
	1200–1450		217–289
HPSC	1200	220±82	
	1300	695±211	
	1400	1860	
	1200–1400		221

a After Costello et al., J. Am. Ceram. Soe. **69** (9), 674–681 (1986).

**Table 6:** Room temperature Weibull parameters, *σ*_0_ and *m* from four-point bend tests of oxidized silicon carbide; SASC = sintered α-SiC; HPSC = hot pressed SiC. *σ*_ave_ is the average bend strength, and *R*^2^ is squared correlation coefficient of the least squares fit to the data^a^

Material	Static load (MPa)	Ox. time (h)	*σ*_ave_ (MPa)	*σ*_0_ (MPa)	*m*	No. of samples	*R*^2^
SASC	0	0.0	575	130	6.8	50	0.97
	0	0.5	600	176	8.3	11	0.95
	0	1.0	580	175	8.4	55	0.95
	0	12.0	560	227	11.4	10	0.92
	0	20.0	555	228	11.5	11	0.93
	0	50.0	570	220	10.7	11	0.95
	160	1.0	500	134	7.7	28	0.98
	330	1.0	540			21	
HPSC	0	0.0	550	177	8.9	49	0.95
	0	0.5	605	218	9.9	11	0.97
	0	1.0	845	279	9.1	49	0.98
	0	12.0	620	252	11.4	11	0.94
	0	20.0	670	361	16.6	10	0.91
	0	50.0	700	502	30.9	10	0.95
	190	1.0	735			15	
	270	1.0	845	277	9.1	15	0.98

a After Easier et al., J. Am. Ceram. Soe. **64**, (12), 731–734 (1981).

**Table 7:** Weibull parameters, *σ*_0_ and *m*, from four-point bend tests of siliconized silicon carbide materials oxidized in dry O_2_ at 1150 °C. *σ*_ave_ is the average bend strength ± one standard deviation, and *R*^2^ is squared correlation coefficient of the least squares fit to the data^a^

Material	Oxidation time (h)	Bend test temperature (°C)	*σ*_ave_ (MPa)	*σ*_0_ (MPa)	*m*	*R*^2^
NC430	0	25	203±15	210	13.7	0.94
	0	1000	213±16	222	12.6	0.94
	0	1150	213±14	220	14.9	0.95
	0	1300	214±9	219	21.1	0.85
	10	1000	234±15	242	15.1	0.96
	10	1150	222±16	230	13.2	0.93
	10	1300	222±12	228	17.7	0.95
	50	1000	221±23	232	9.2	0.99
	50	1150	224±17	233	12.5	0.79
	50	1300	228±17	237	13.3	0.97
HD530	0	25	165±13	172	12.6	0.99
	0	1000	170±16	178	10.4	0.95
	0	1150	179±18	187	9.6	0.96
	0	1300	190±17	198	11.2	0.94
	10	1000	161±11	167	14.8	0.98
	10	1150	171±12	177	13.9	0.87
	10	1300	195±8	199	24.6	0.96
	50	1000	58±6	161	25.9	0.97
	50	1150	174±13	180	12.8	0.94
	50	1300	189±13	195	13.7	0.96
CS101K	0	25	224±44	243	5.3	0.95
	0	1000	234±14	242	14.6	0.87
	0	1150	215±15	223	14.0	0.98
	0	1300	225±13	231	16.3	0.80
	10	1000	242±18	250	13.1	0.95
	10	1150	213±19	223	10.8	0.99
	10	1300	232±8	236	27.6	0.92
	50	1000	244±17	257	8.6	0.97
	50	1150	222±17	230	12.9	0.95
	50	1300	253±17	262	13.0	0.75

a After Y. Tsai et al., EM-5274, Final Report for Research Project 2260-2, Electric Power Research Institute (1987).

**Table 8:** Active corrosion of pressureless sintered SiC under-the linear eorrosion kineties of a salt at 900 °C and 100 kPa (1 atm) of gas^a^

Gas	Salt	Corrosion rate (mg · cm^−2^ · h^−1^)
Air	Na_2_SO_4_	−17
Air	Na_2_SO_4_ · 5% C	−29
Air	Na_2_SO_4_ · 10% C	−28
Air	Na,SO_4_ · l% NaNO_3_	−34
Air	Na_2_S0_4_ · l% Na_2_O	−37
Air	Na_2_SO_4_ · l% Na_2_S	−35
Air	Na_2_SO_4_ · l% Na_2_CO_3_	−34
Air	Na_2_SO_4_ · 5% Na_2_CO_3_	−39
O_2_	Na_2_SO_4_ · l% Na_2_CO_3_	−18
O_2_	Na_2_S0_4_ · 2% Na_2_CO_3_	−16
O_2_	Na_2_SO_4_ · 10% Na_2_CO_3_	−17
O_2_	Na_2_CO_3_	−45

a After D. W. MeKee et al„ J. Am. Ceram. Soe. **59** (9–10), 441–444 (1976).

**Table 9:** Four-point bend strength (± 1 standard deviation) of α-SiC (Hexoloy) after exposure to a salt corrodent at 1000 °C for 48 h^a^

Gas	Salt	Average strength (MPa)
Air	None	409±62
Air	Na_2_SO_4_	251±45
SO_3_	Na_2_SO_4_	207±72
CO_2_	Na_2_CO_3_	355±70

a After J. L. Smialek et al., J. Am. Ceram. Soc. **69** (10), 741–752 (1986).

**Table 10:** Surface recession rates for commercial SiC materials exposed to industrial furnace environments. A number greater than zero indicates that a deposit grew on the surface^a^

Furnace environment	Maximum temperature (°C)	Recession Hexoloy SA	rate NC400	(mm/yr) NC430
Aluminum remelt No. 1	1040	−0.28	−0.14	−0.42
Aluminum remelt No. 2	1150		−3.78	−1.89
Steel soaking pit No. 1	925	−0.05	−0.03	−0.17
Steel soaking pit No. 2	1250	−3.53		−4.32
Steel reheat	1100	−0.05		−0.69
Forge	1175	+ 0.05	+ 0.31	−0.13

a After J. I. Federer et al., Adv. Ceram. **14**, 315–334 (1985).

**Table 11:** Surface recession rates for various SiC materials exposed to industrial furnace environments^a^

Furnace environment	Temperature range (°C)	Recession rate (mm/yr)			Hot pressed
		Hexoloy SA	CS101	CS101K	
Aluminum remelt No. 1	750–1250	−2.8	−7.15	−7.00	−7.00
Aluminum remelt No. 2	750–1200		−2.07	−1.94	−1.12
Steel soaking pit	1150–1175	−0.4	−0.90	−0.15	−0.15

a After J. I. Federer, Ceram. Trans. **10**, 425–443 (1989).

**Table 12:** Room temperature fracture strength of Hexoloy SA determined by c-ring tests after exposure to industrial furnace environments

Furnace environment	Number of samples	Average strength (MPa)
aAir	18	280±50
aAluminum remelt	12	243±23
bAluminum remelt No. 1	12	213±39
bSteel soaking pit	11	246±56
bSteel reheat	12	221±34
bForge	9	286±43

a After J. Luyten et al., High Temperature Corrosion of Technical Ceramics (1900) pp. 161–168.

b After J. I. Federer et al., Adv. Ceram. 14, 315–334 (1985).

**Table 13:** Simulated gas composition for a medium-energy coal gasification environment^a^

Gas:	H_2_	CO	CO_2_	N_2_	H_2_S	H_2_O
Vol. %	30	44	10	1.4	0.6	14

a After T. E. Easier et al., in High Temperature Corrosion in Energy Systems (1985) pp. 269–280.

**Table 14:** Composition (percent by weight) of various coal slags

Component	Slag 1^a^	Slag 2^b^	Slag 3^c^	Slag 4^c^	Slag 5^d^	Slag 6^d^
SiO_2_	45.77	50.80	50	30	54	56
Fe_2_O_3_	10.72	16.28	25	15	21	4
Al_2_O_3_	14.17	23.93	20	10	19	31
CaO	18.75	2.72	5	25	0.1	0.5
MgO	7.08	0.72		20	0.9	0.8
NiO	0.01					
SO_3_	0.055	1.65				
P_2_O_5_	0.51	0.27				
Na_2_O	1.10	0.47			0.6	2.5
K_2_O	0.52	1.60			1.7	2.6
TiO_2_	0.27	0.62			1.3	0.8
SrO + BaO	0.16					
V_2_O_5_	0.03					
Base/Acid	0.63	0.29	0.43	1.5	0.33	0.12

a After M. K. Ferber et al., J. Am. Ceram. Soe. **68** (4), 191–197

(1985).

b After M. K. Ferber et al., Ceram. Bull. **62** (2), 236–243 (1983).

c After T. E. Easier et al., in High Temperature Corrosion in Energy Systems (1985) pp. 269–280.

d After P. F. Becher, J. Matls. Sei. **19**, 2805–2814 (1984).

**Table 15:** Corrosion rates for various commercial grades of SiC exposed to various environments for 200 h at 1250 °C. (See Table 14 for slag compositions)^a^

Material	Condition	No slag (air) (μg · cm^−2^ · h^−1^)	Acidic slag (Slag 3) (μg · cm^−2^ · h^−1^)	Basic slag (Slag 4) (μg · cm^−2^ · h^−1^)
Hexoloy SA	Slip-cast	+ 0.7	−174	−657
Hexoloy SA	Extruded	−0.4	− 50	−412
NC430	Slip-cast	+1.6	−117	−412
NC430	Extruded	+ 5.7	− 50	−136

a After T. E. Easier et al., in High Temperature Corrosion in Energy Systems (1985) pp. 269–280.

**Table 16:** High-temperature c-ring fracture strength of Hexoloy SA SiC after exposure to various environments. (See Table 14 for the slag composition.)^a^

Environment	Temperature (°C)	Exposure time (h)	Fracture strength (MPa)
Air	1200	0	397±7.6
Air	1200	24	375±6.6
Air	1300	0	354±8.9
Air	1300	24	361±9.3
Slag 1	1200	24	335±9.3
Slag 1	1300	24	285±3.6

a After M. K. Ferber et al., J. Am. Ceram. Soe. **68** (4), 191–197 (1985).

**Table 17:** Four-point flexural strengths of silicon carbide materials after exposure to various environments. Materials were exposed to the slag for 1150 °C for 350 h. (See Table 14 for the composition of the slag.)^a^

Material	Environment	Temperature	Flexural strength (MPa)
NC203	Air	22	707
NC203	Air	1000	621
NC203	Air	1200	550
NC203	Air	1300	493
NC203	Slag 6	22	386
NC203	Slag 6	1000	414
NC203	Slag 6	1150	429
NC203	Slag 6	1300	371
Hexoloy SA	Air	22	407
Hexoloy SA	Air	1000	464
Hexoloy SA	Air	1300	471
Hexoloy SA	Slag 6	22	286
Hexoloy SA	Slag 6	1150	343
Hexoloy SA	Slag 6	1300	429
KT	Air	22	200
KT	Slag 6	22	186
KT	Slag 6	1150	221
KT	Slag 6	1300	214

a After P. F. Bccher, J. Matls. Sci. **19**, 2805–2814 (1984).

**Table 18:** Molecular oxygen diffusivitics of silica and silicon oxynitride^a^

Material	Temperature (°C)	Diffusivity (10^−8^ cm^2^/s)
SiO_2_	1100	1.42
SiO_2_	1200	2.78
SiO_2_	1300	5.00
SiO_2_	1400	8.37
Si_2_N_2_O	1200	0.00240
Si_2_N_2_O	1300	0.00927
Si_2_N_2_O	1400	0.110

a After R. E. Tressler et al., in High Temperature Corrosion of Technical Ceramics (1990), pp. 69–89.

**Table 19:** Composition of the oxide scale formed on hot-pressed HS-130^a^

Temperature (°C)	Exposure time (h)	O_2_ Pressurte (kPa)	Si (wt%)	Mg (wt%)	O (wt%)
1300	24	80	27.4 ± 2	17.37 ± 2.8	55.2
1350	194	20	28.1 ± 0.8	16.8 ± 0.5	55.12
1400	24	10	28.0 ± 0.3	23.15 ± 0.8	48.89
1400	24	20	29.0 ± 3.5	22.0 ± 4.0	49.0
1400	24	40	27.9 ± 0.5	21.61 ± 0.8	50.53
1400	24	80	27.9 ± 0.5	21.96 ± 0.5	50.15
1450	21	20	30.0 ± 0.6	26.68 ± 1.5	46.26
1475	24	20	28.6 ± 0.5	21.9 ± 1.5	49.49
1500	24	20	39	10	51

a After W. C. Tripp et al., J. Am. Ceram. Soc. 59 (9–10), 399–403 (1976).

**Table 20:** Active-to-passive transition temperature, *T*_ap_, for hot pressed HS-130 and sintered 6Y-14 silicon nitride at various values of the partial pressure of oxygen, *P*(O_2_).^a^

Material	Flow rate (em^3^/s)	*P*(O_2_) (Pa)	*T*_ap_ (°C)
HS-130	0.56	7.1	1366
HS-130	0.56	27.4	1436
HS-130	0.56	205.5	1520
6Y-14	0.56	7.0	1365
6Y-14	0.56	28.5	1446
6Y-14	0.56	111.0	1480

a After W. L. Vaughn et al., J. Am. Ceram. Soc. 73 (6), 1540–1543 (1990).

**Table 21:** The room temperature Weibull paramerters for sintered SNW-1000 and hot-pressed NCX-34 silicon nitride ceramics after oxidation at 1370 °C in air under various loads. *σ*_0_ = Weibull characteristic strength, *m* = WeibuII modulus, and *R*^2^ = correlation coefficient^a^

Material	Load during oxidation (MPa)	Oxidation time (h)	Number of samples (MPa)	*σ*_0_	*m*	*R*_2_
SNW-1000	0	0	50	300	8.2	0.98
SNW-1000	0	0.5	10	325	7.7	0.97
SNW-1000	0	1	53	290	10.9	0.94
SNW-1000	0	12	11	275	17.2	0.97
SNW-1000	0	20	11	215	8.3	0.90
SNW-1000	0	50	10	225	19.6	0.98
SNW-1000	23	1	15	390	10.2	0.99
SNW-1000	45	1	15	295	7.0	0.97
NCX-34	0	0.5	10	555	7.0	0.96
NCX-34	0	1	54	575	14.1	0.96
NCX-34	0	12	11	400	7.0	0.92
NCX-34	0	20	12	500	14.3	0.96
NCX-34	0	50	12	435	7.6	0.94
NCX-34	45	1	14	490	9.7	0.93
NCX-34	158	1	13	385	7.9	0.93

a After T. E. Easier et al., J. Am. Ceram. Soc. 65 (6), 317–320 (1982).

**Table 22:** Parabolic rate parameter, *K*_p_, for the oxidation of hot-pressed HS-130 silicon nitride in O_2_ with a partial pressure of water vapor of 3.3 kPa (0.033 atm)^a^

Temperature (°C)	log *K*_p_ (g^2^•cm^−4^•s^−1^)
1200	−12.6
1260	−12.0
1315	−11.3
1370	−10.8

a After S. C. Singhal, J. Am. Ceram. Soc. 59 (1–2) 81–82 (1976).

**Table 23:** Room-temperature flexural strength, *σ*, of hot isostatically pressed AY6 silicon nitride after exposure at 1400 °C for 10 h in H_2_ atmospheres with various partial pressures *P*(H_2_O) of water vapor^a^

*P*(H_2_O) (Pa)	σ (MPa)
0.26	580 ± 35
0.45	525 ± 60
3.6	515 ± 30
20	460 ± 40
100	690 ± 60
520	800 ± 80

a After H. Kim et al., Ceramic Transactions 10, 81–96 (1989).

**Table 24:** Four-point bend strengths of hot-pressed silicon nitride materials after exposure to an aluminum remelting furnace^a^

Sintering Aid	Exposure time (h)	Number of samples	Average strength (MPa)
Y_2_O_3_	0	20	610±39
Y_2_O_3_	100	8	437±62
MgO	0	10	610±54
MgO	100	8	282±38

a After J. Luyten et al., High Temperature Corrosion of Technical Ceramics (1990) pp. 161–168 (1990).

**Table 25:** Room-temperature four-point flexural strengths of silicon nitride materials after exposure to various environments. (See Table 14 for the composition of the slag.)^a^

Material	Environment	Temperature (°C)	Exposure time (h)	Flexural strength (MPa)
NC-132	Air	22	0	850
NC-132	Slag 5	1220	500	325
NC-132	Slag 6	1150	350	430
NCX-34	Air	22	0	810
NCX-34	Slag 5	1220	500	300
NCX-34	Slag 6	1150	350	325

a After P. F. Becher, J. Matls. Sci. 19, 2805–2814 (1984).

**Table 26:** The partial pressures of oxygen, $P_{O_{2}}$, above which selected oxides are stable. The concurrent partial pressure of sulfur, *P*_S_, is also shown^a^

Oxide	*T* = 1150 K log_10_ ( $P_{O_{2}}$) (*P* in Pa)	(877 °C) log_10_(*P*s) (*P* in Pa)	T=1450 K log_10_ ( $P_{O_{2}}$) (*P* in Pa)	(1177 °C) log_10_ (*P*_S_) (*P* in Pa)
SiO_2_	−27	0	−18	2
Al_2_O_3_	−34	13	−24	− 8
ZrO_2_	−35	−17	−25	−11
Ce_2_O_3_	−40	−31	−28	−21
Cr_2_O_3_	−20	− 7	−13	− 3
MgO	−39	−23	−26	−15

a After E. A. Gulbranson and G. H. Meier, DOE/FE/13547-01, UC-90h (May 1980).

**Table 27:** Burner rig corrosion test results of thermal barrier coatings^a^

Coating	Intermediate layer	Overcoat	Time to failure (h)
PS ZrO_2_	NiCrAlY Bond Coat	Pt	242
PS ZrO_2_	NiCrAlY Bond Coat	AlPt	>500
PS ZrO_2_	NiCrAlY Bond Coat + ZrO2/NiCrAiY	Pt	>500
PS ZrO_2_	NiCrAlY Bond Coat + ZrO2/NiCrAlY	AlPt	500
PVD ZrO_2_	none	none	22–70

a C. A. Anderson et al., NASA Report CR-65919 (February, 1982).

Notes:
PS = Plasma spray Bond Coat = 127 μm thick ZrO_2_ = 380 μm thick PVD = Electron Beam Physical Vapor Deposition ZrO_2_/NiCrAlY = 50 vol% ZrO_2_−8Y_2_O_3_ + 50 vol% NiCrAlY Pt: 31 μm thick sputter deposited AlPt: 14 μm thick sputter deposited

Test Conditions:
Fuel: No. 2 Distillate with:
ElementNaMgClKCaSAmount(ppm)10010180342 Nominal gas temperature = 1204 °C Maximum metal temperature = 843 °C Test duration=500 h Cycle = 55 min. heating + 5 min. air cooling

## Footnotes

1 Acidic components are taken as SiO_2_, Al_2_O_3_, and TiO_2_, while basic components are Fe_2_O_3_, CaO, MgO, Na_2_O, and K_2_O.
